# The role of interventional radiology and molecular imaging for near infrared photoimmunotherapy

**DOI:** 10.1007/s11604-024-01567-7

**Published:** 2024-04-25

**Authors:** Hisataka Kobayashi, Peter L. Choyke

**Affiliations:** Molecular Imaging Branch, Centre for Cancer Research, National Cancer Institute, NIH, 10 Centre Drive, Bethesda, MD 20892 USA

**Keywords:** Near-infrared photoimmunotherapy, Image-guided intervention, CT, ^18^F-FDG PET, Fluorescence imaging

## Abstract

Near infrared photoimmunotherapy (NIR-PIT) is a recently approved cancer therapy for recurrent head and neck cancer. It involves the intravenous administration of an antibody-photoabsorber (IRDye700DX: IR700) conjugate (APC) to target cancer cells, followed 24 h later by exposure to near infrared light to activate cell-specific cytotoxicity. NIR-PIT selectively targets cancer cells for destruction and activates a strong anticancer host immunity. The fluorescent signal emitted by IR700 enables the visualization of the APC in vivo using fluorescence imaging. Similarly, the activation of IR700 during therapy can be monitored by loss of fluorescence. NIR-PIT can be used with a variety of antibodies and therefore, a variety of cancer types. However, in most cases, NIR-PIT requires direct light exposure only achieved with interstitial diffuser light fibers that are placed with image-guided interventional needle insertion. In addition, the unique nature of NIR-PIT cell death, means that metabolic molecular imaging techniques such as PET and diffusion MRI can be used to assess therapeutic outcomes. This mini-review focuses on the potential implications of NIR-PIT for interventional radiology and therapeutic monitoring.

## Introduction

Three major cancer therapies; surgery, radiation and chemotherapy, have been mainstays in oncologic therapy since the beginning of modern medicine. More recently, immunotherapy, such as immune-activating cytokine therapy, checkpoint inhibition, engineered T-cells and suppressor cell depletion, have been added to this list. Although immunotherapies can produce dramatic results, unfortunately, a minority of patients respond. Simultaneously destroying cancer cells and activating anticancer host immunity with one treatment has been a long-hoped-for dream. Here, we describe the use of a particular hydrophilic photo-absorbing dye based on the silicon-phthalocyanine derivative, IRdye700DX (IR700), which is covalently conjugated to a cancer-targeting antibody (mAb). When this conjugate binds to a tumor and is exposed to low dose near infrared light, fluorescence imaging can demonstrate accumulation. As the light dose is increased highly selective and rapid cell death occurs, a process known as “near infrared photo-immunotherapy” (NIR-PIT) [1]. NIR-PIT begins with the intravenous administration of an antibody-photoabsorber conjugate (APC: mAb conjugated with IR700). About a day later NIR laser light is applied to the tumor and cytotoxicity begins within 1 min of light exposure. The nature of the induced cell death is known as necrotic/immunogenic cell death [2] because it involves the rapid release of both cancer antigens and immune activation signals that engage the host immune system [3]. Meanwhile, a very high therapeutic index is achieved because virtually no phototoxicity is seen in adjacent antigen-negative normal cells. In preclinical studies, 80% of immunocompetent mice showed tumor-free survival with optimized regimens, especially when combined with immune-activation therapies including immune checkpoint inhibitors [3] or immune-suppressive cell targeted NIR-PIT [4, 5].

**Fig. 1:**
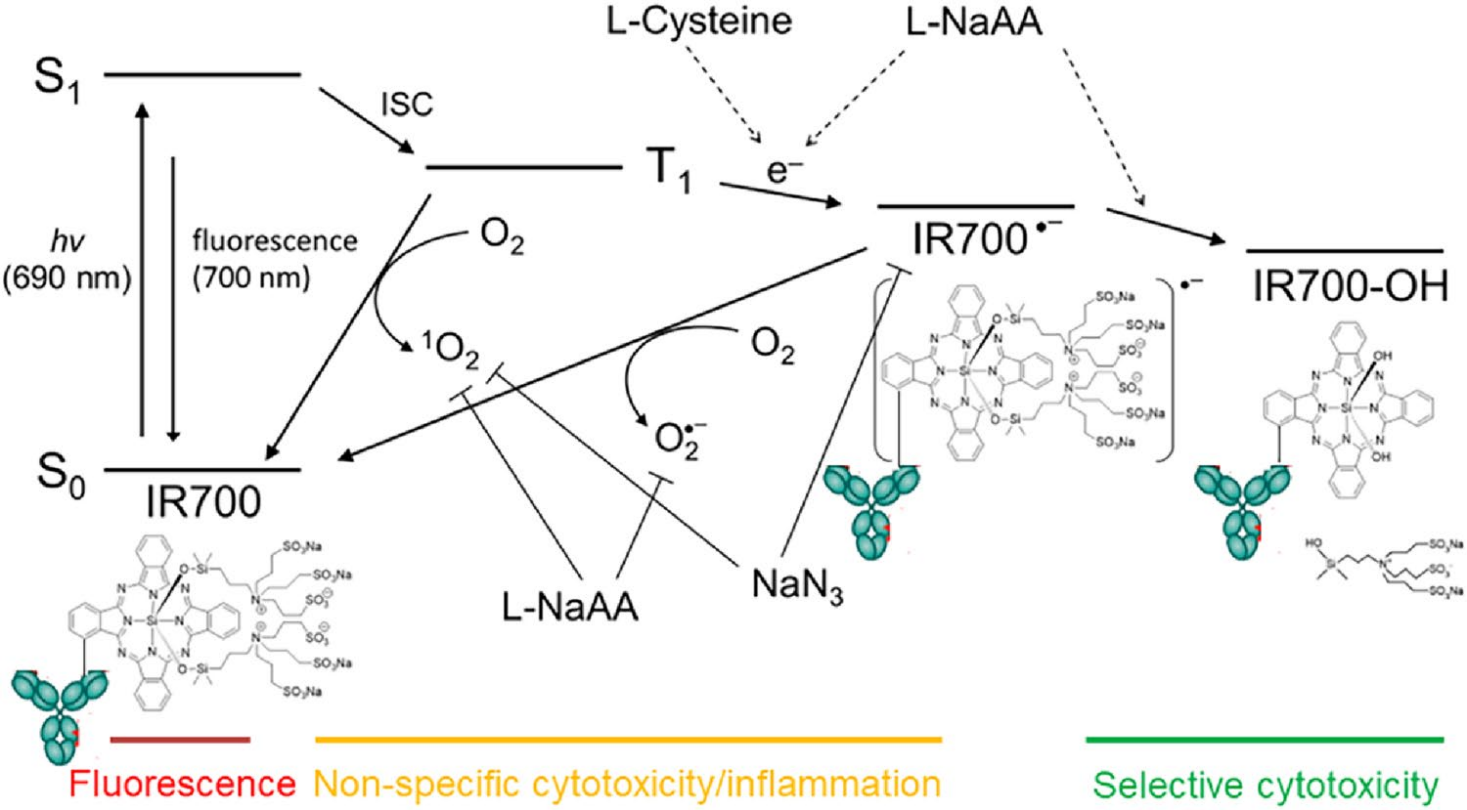
A diagram of IR700 photo-chemical reaction for near infrared photoimmunotherapy. A diagram of photo-chemical reaction explains selective cytotoxic mechanism of near infrared photoimmunotherapy (NIR-PIT) that shows differences between photo-dynamic therapy and NIR-PIT

NIR-PIT was effective only when conjugates were bound to the cell membrane; unbound APCs elicited no phototoxicity. This is due to the unique mechanism of action of NIR-PIT in which rapid photo-induced ligand release occurs leading a profound change in chemical properties in the APC which in turn, leads to membrane damage [6, 7]. This mechanism is to be contrasted with conventional photodynamic therapies (PDT) that rely on oxidation with reactive oxygen species and have far more off target effects (Fig. 1).

Successful preclinical NIR-PIT studies against cancer and cancer-stem like cell antigens have been performed against more than 20 different molecular targets expressed on different cancers including EGFR, HER2, PSMA, CD25, GPC3, mesothelin, CD133, CD44, CEA, DLL3, PD-L1, and others [8]. Indeed, the list of viable antibodies for NIR-PIT continues to grow underscoring the potential flexibility of the method. So long as the antigen is robustly expressed on the cancer cell surface, NIR-PIT will be successful in killing cancer cells and inducing a host immune response (provided the immune system is intact). In addition, NIR-PIT can also be directed against immune-suppressive cells in the tumor microenvironment, for example, anti-CD25 [5], CTLA-4 [9], VISTA [10], Ly6G [11], CD206 [12] can be used with NIR-PIT to amplify the immune effect induced by the therapy [13]. Four antibodies targeting EGFR, CD25, PSMA, and PD-L1, all of which are targeting cancers, yet anti-CD25 can also target Treg cells and anti-PD-L1 can block immune checkpoint for further enhancing anticancer host immunity [5, 14], have progressed to the clinical application stage but many more are possible in the future.

Currently an FDA-designated fast-track global phase 3 trial is ongoing world-wide including in the US, EU and Asia. This trial uses the EGFR-targeting NIR-PIT drug (cet-IR700; Akalux^™^) and a NIR laser (Bioblade^™^) for NIR-PIT. The trial treats recurrent head and neck cancers with NIR-PIT. Akalux and Bioblade were approved for clinical use in Japan in September 2020. Since then, NIR-PIT has been performed more than 400 times in over 200 patients in more than 130 hospitals in Japan [15].

**Fig. 2:**
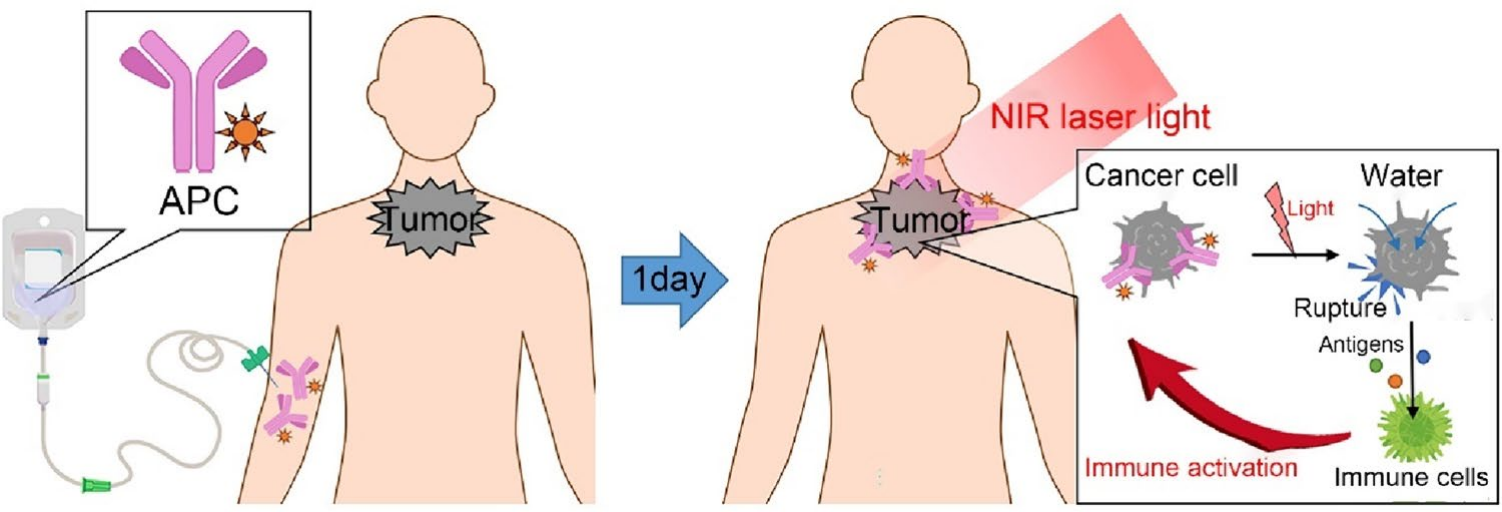
Procedures of NIR-PIT. For NIR-PIT procedures, patients undergo intravenous drip infusion of APC for 2 h on the day prior to NIR light exposure. The following day, tumors are exposed to either 50 J/cm^2^ or 100 J/cm of 690 nm NIR laser light

For NIR-PIT procedures, patients undergo intravenous drip infusion of APC for 2 h on the day prior to NIR light exposure. The following day, tumors are exposed to either 50 J/cm^2^ or 100 J/cm of 690 nm NIR laser light. This exposure is achieved with the use of a frontal light diffuser for surface lesions or with an interstitial light diffuser inserted into the tumor for deeper lesions. (Fig. 2). The penetration of NIR light is approximately 1 cm which necessitates placement of interstitial light diffusers for lesions deeper or wider than that.

Because NIR-PIT necessitates localized light exposure to tumors, image-guided interventional procedures are essential for accurately placing fiberoptic diffusers to ensure effective therapy. One advantage of NIR-PIT is that light can be applied so that it overlaps both normal and tumor tissue since the damage to normal tissue is nil. Nonetheless, adequate coverage of the entire tumor is needed to maximize the success of NIR-PIT. Because NIR-PIT induces a unique process of cell death compared to conventional cancer therapies, appropriate image-based monitoring of NIR-PIT is necessary. In this mini-review, the potential contributions of radiological technologies to NIR-PIT in the clinical setting are discussed.

## Image-guided interventional procedures for accurately placing fiber optic diffusers

**Fig. 3:**
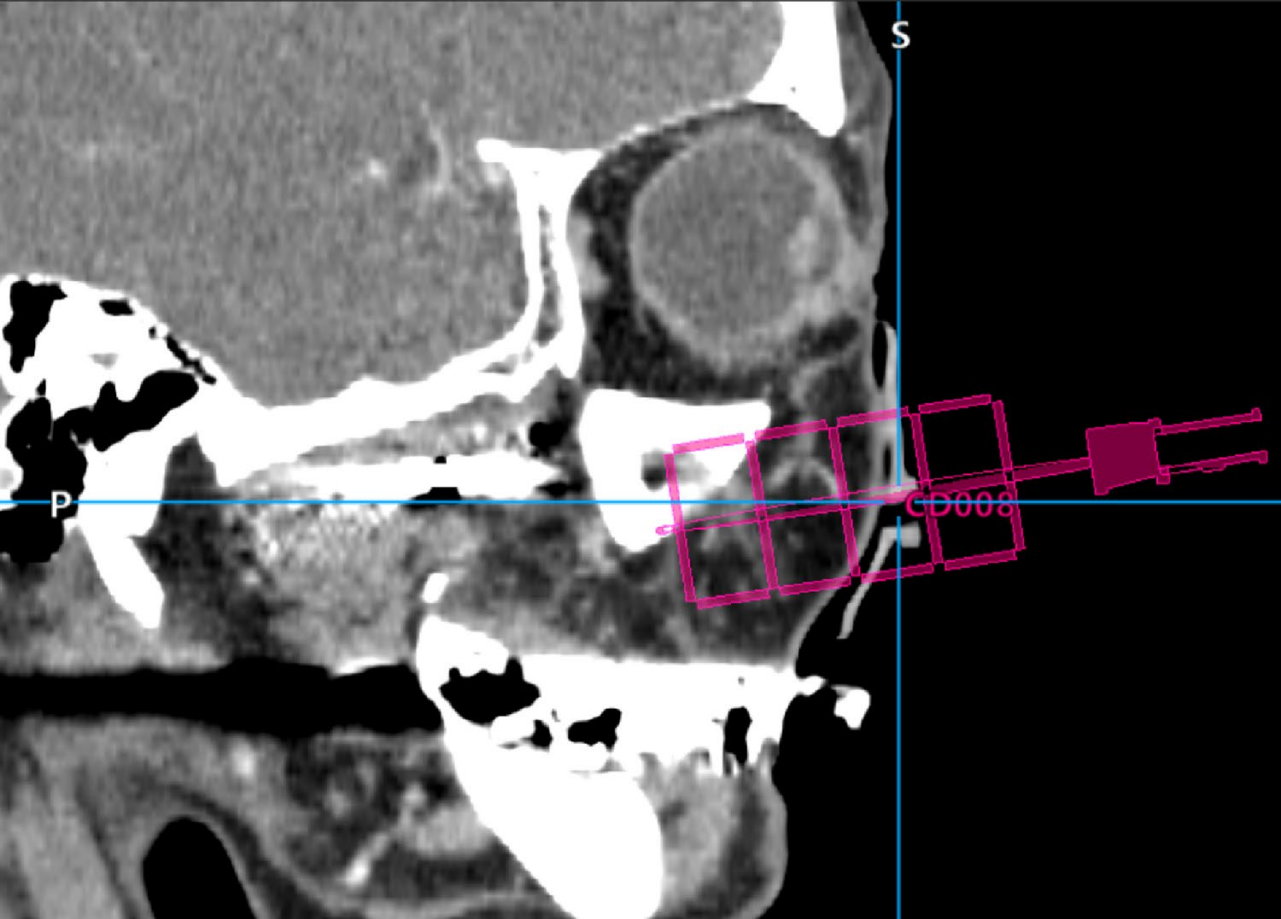
A sagittal reconstructed CT navigation image for NIR-PIT needle insertion. (Courtesy of Prof. Takayoshi Suzuki, Hokkaido University, Graduate School of Medicine)

After APCs bind to target cancer cells, NIR light is used to activate the photo-absorber. Imaging methods such as CT, US, MRI and fluorescence imaging can be used to accurately place guide needles through which the interstitial fiber optics can be inserted. Image-guided navigation for needle insertion is already a common practice in NIR-PIT [16]. For instance, in patients undergoing NIR-PIT for head and neck cancer several CT navigation systems have been employed (Fig. 3). In another current clinical trial, which uses NIR-PIT in the setting of metastatic liver tumors, regulatory T-cells are depleted with antiCD25 (basiliximab)-IR700 NIR-PIT (RM-1995) after needle insertion under CT- or US guidance.

New optical fibers have been developed that are suitable for intra-arterial applications via intra-vascular catheters inserted using the Seldinger technique. Given that the current light fibers have a diameter of 1 mm, comparable to the diameter of guide wires used in angiography, this approach potentially offers a less invasive means of accessing tumors as compared to direct needle insertion.

Thus, it is already clear that imaging guidance will play a major role in the accurate placement of interstitial fibers within target tumors. One challenge is to ensure that adequate light dosing (light dosimetry) is achieved throughout the entire tumor and software is currently being developed to accomplish this goal.

## Fluorescence-guided imaging for diagnosis and treatment monitoring

**Fig. 4:**
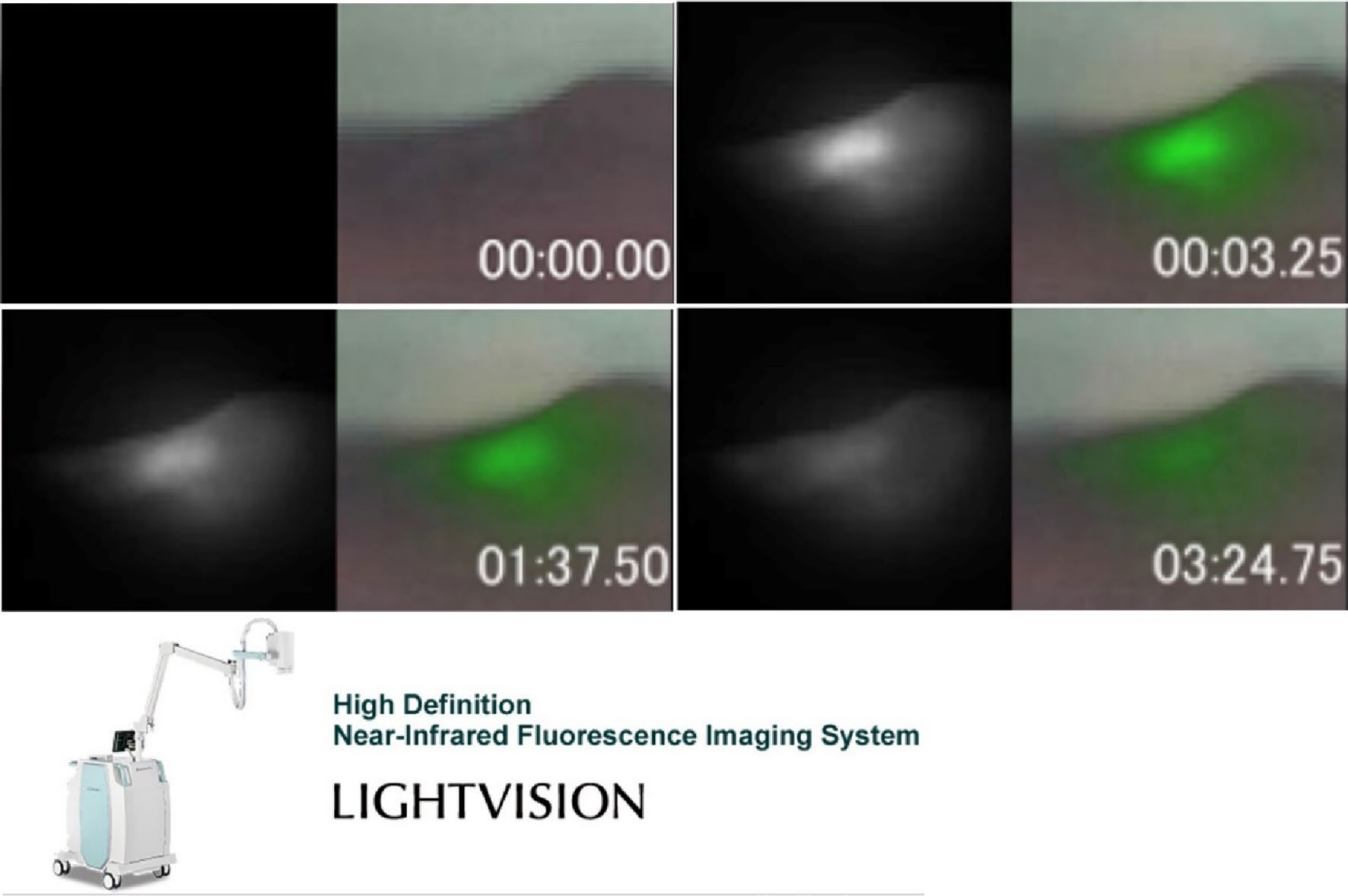
Serial fluorescent images during NIR-PIT. Serial fluorescent images during NIR-PIT were obtained by Lightvision (Now LuminousQuester NI, Shimadzu Inc, Kyoto Japan). IR700 fluorescence in the tumor was disappearing as NIR light exposure

The IR700 dye emits fluorescence with sufficient strength to be detected by an appropriate camera, enabling fluorescence imaging to visualize the accumulation of APC in tumor beds. This feature can be useful in guiding light treatment [17]. In addition, during therapeutic light exposure, IR700 releases axial ligands that induce the formation of Z-stacked dimers, resulting in a loss of fluorescent emission (Fig. 4). Therefore, by monitoring IR700 fluorescence produced by the APC one can not only detect the target tumor, but also monitor the efficacy of therapy in real time. The disappearance of IR700 fluorescence indicates the consumption of IR700 during NIR-PIT and demonstrates that the tumor has received sufficient exposure of NIR light to induce therapeutic effects. To facilitate this in a clinical setting, we have developed a NIR fluorescence camera system in collaboration with Shimadzu Inc. and are currently conducting a clinical trial to demonstrate its efficacy.

## Metabolic and molecular imaging for monitoring early therapeutic effects of NIR-PIT

**Fig. 5:**
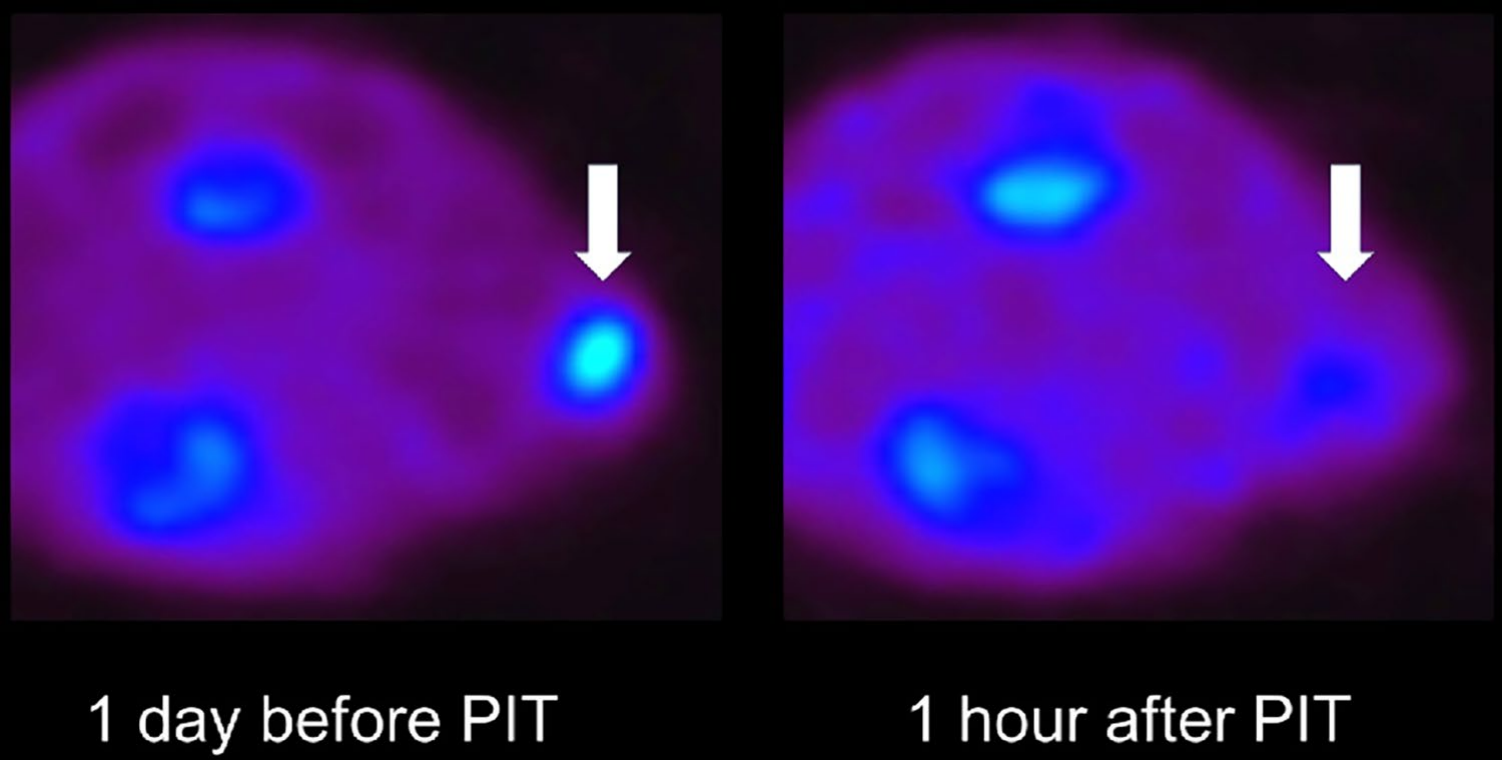
^18^F-FDG PET images before and 1 h after NIR-PIT. ^18^F-FDG uptake in experimental A431 tumor decreased over 90% 1 h after NIR-PIT targeting EGFR employing cetuximab-IR700 that is the same formula of clinically approved Akalux^™^ (Rakuten Medical Inc. San Diego, CA)

NIR-PIT induces a pure immunogenic cell death in targeted cancer cells by damaging cellular membranes within a few minutes after exposure of NIR light. Dying cells release large amounts of ATP that effectively stops all metabolic activity. Normally, cancer cells exhibit higher uptake of glucose when compared with normal cells, a process efficiently visualized by ^18^F-FDG PET. Theoretically, this glucose metabolism stops immediately after NIR-PIT. In preclinical animal experiments, ^18^F-FDG PET demonstrated greater than 90% cancer cell death in vivo immediately after NIR light exposure, a finding that was consistent with loss of signal on ATP-dependent bioluminescence imaging [18]. (Fig. 5) The results of early ^18^F-FDG PET after NIR-PIT in head and neck squamous cell cancer patients was reported in the Society of Nuclear Medicine and Molecular Imaging meeting in 2023. When obtaining early ^18^F-FDG PET scans within a day after NIR-PIT, some acute inflammatory reaction is expected to result in mild uptake of FDG, but overall, there was distinct decrease in metabolic activity in the tumor within 24 h after NIR-PIT despite no apparent change in tumor size.

Diffusion MRI was able to depict early changes after NIR-PIT; however, the Apparent Diffusion Coefficient (ADC) values varied depending on the timing after NIR-PIT [19]. In brief, the ADC values decreased immediately after NIR light exposure, reflecting the release of massive intracellular contents, followed by a gradual increase in ADC indicative of increased free water in the interstitial space. Consequently, utilizing diffusion MRI as an early biomarker may be challenging.
